# The Environment Matters: Perspectives on Using the Physical Environment in a Skilled Nursing Facility for Clients with Dementia

**DOI:** 10.1002/alz.089985

**Published:** 2025-01-09

**Authors:** Jennie L DiGrado

**Affiliations:** ^1^ University of St. Augustine for Health Sciences, San Marcos, CA USA

## Abstract

Occupational therapists (OTs) are among the health care professionals who assist individuals with neurocognitive disorders (NCDs) to engage in self‐care and leisure activities in a skilled nursing facility (SNF) environment. OTs use various environmental modifications to help individuals with NCDs engage in various activities (Jensen & Padilla, 2017). Strong evidence has been found to support occupational therapy recommendations and interventions in adapting the physical environment to support person centered care (Gitlin et al., 2010; Jensen & Padilla, 2017; Zimmerman et al., 2013). This session aims to share findings from a qualitative case study regarding the perspective of OTs and how they use the built physical environment to help individuals with NCDs is a SNF engage in self‐care and leisure related activities. Recommendations from this study include nonpharmacological‐based intervention strategies that incorporate the use of the built environment in a SNF to improve the quality of life for clients with a NCD.

